# Medical Society of the State of Georgia

**Published:** 1859-04

**Authors:** Eben Hillyer

**Affiliations:** Secretary


### EDITORIAL AND MISCELLANEOUS.

### MEDICAL SOCIETY OF THE STATE OF GEORGIA.

The next Annual Meeting of this Body, will be held in the city of Atlanta, on the second Wednesday, the 13th of April next. EBEN HILLYER, Secretary.

We publish the above, the official notice of the annual meeting of the Medical Society of the State, to which attention has been already directed through this journal.

We would again urge the medical men of the State, to attend, and assure them that they will meet with a most cordial welcome from their Atlanta brethren. As an additional inducement for a large attendance, we would state that, besides the scientific and ordinary professional interest always connected with the annual meeting of the society, the profession of the city have projected a series of social entertainments, which we think will add greatly to the pleasure of the occasion.

